# No time to waste: necessary health support for retired professional rugby players

**DOI:** 10.17159/2078-516X/2021/v33i1a10651

**Published:** 2021-01-15

**Authors:** V Gouttebarge, DC Janse van Rensburg, GM Kerkhoffs

**Affiliations:** 1 Amsterdam UMC, University of Amsterdam, Department of Orthopaedic Surgery, Amsterdam Movement Sciences, Meibergdreef 9, Amsterdam, the Netherlands; 2 Section Sports Medicine, Faculty of Health Sciences, University of Pretoria, Pretoria, South Africa; 3 Amsterdam Collaboration on Health & Safety in Sports (ACHSS), Amsterdam UMC IOC Research Center of Excellence, Amsterdam, the Netherlands; 4 Academic Center for Evidence-based Sports Medicine (ACES), Amsterdam, the Netherlands

**Keywords:** rugby, transitioning, long-term health, health surveillance

## Abstract

There has been increasing scrutiny of professional rugby following the concern that retired players might face several negative health conditions. Currently, support measures addressing the health of retired professional rugby players are not systematically implemented. This is unusual as professional rugby stakeholders have the duty of care to protect and promote the long-term health of retired players. Professional football has a health programme for retired players that is implemented globally. This programme formed the basis for the After Rugby Career Consultation (ARCC) which was developed to empower the sustainable health and quality of life of retired professional rugby players. The ARCC relies on information from three sources: (1) educational material, (2) medical examination, and (3) guidance, referral and/or monitoring. The South African rugby stakeholders have connected to pilot the ARCC as there is no time to waste: a step towards necessary health support for retired professional rugby players is needed.

Transitioning out of professional sport is not an easy process, for example, athletes might face several challenges, such as adjusting to a new lifestyle, being suddenly ‘like everyone else’, and missing the sports atmosphere and competition. In addition, transitioning athletes might also face several health conditions affecting various spheres of the body and personality (e.g. musculoskeletal, psychological, neurocognitive). Rugby union (hereafter referred to as ‘rugby’) has been associated with various post-career health conditions, such as osteoarthritis (OA) and mental health symptoms. As with other high-speed collision sports, professional rugby has been considerably under scrutiny when it comes to the long-term health of players. Despite the growing body of scientific evidence and many recent anecdotal reports of retired players facing health challenges, support measures addressing the health of retired professional rugby players from a holistic perspective are not systematically implemented yet. Neither is there any guidance for either the prevention of long-term health conditions or the promotion of their remission. The objective of this article is to reflect on the concept of long-term health in professional rugby and to introduce the After Rugby Career Consultation (ARCC) as a support measure for retired professional rugby players.

## Health challenges after retirement from professional rugby

Scientific evidence shows that professional rugby players might be exposed to various health challenges after retirement from the game. OA is the most common rheumatic disease worldwide resulting in joint pain and activity limitations. In professional and well-trained rugby players, OA results from a complex interaction of biological, mechanical, and biochemical factors. In most cases it is precipitated by traumatic or overuse injuries that accelerate intra-articular pathological processes. Consequently, retired professional rugby players may have an earlier onset and a higher prevalence of OA than the general population (matched for age and gender). For instance, two recent studies showed that the prevalence of ankle and hand OA in retired professional rugby players reached five percent (higher than that of the general population). Ten percent of these retired players specifically reported hand pain.^[1,2]^ Mental health symptoms, defined as negative thoughts, feelings and/or behaviours, are also commonly reported following a professional rugby career. A study conducted on nearly 300 retired professional rugby players (mean age of 38 years) found prevalence rates ranging from 24% for alcohol misuse to 29% for sleep disturbances.^[3]^ These prevalence rates are similar to those found among former elite athletes from other sports, but seem slightly higher than those found among the general population.^[3,4]^ Players forced to retire (e.g. due to a career ending injury) were more likely to report mental health symptoms in comparison to those that retired voluntarily.^[5]^ When it comes to neurocognition, recent research has suggested an association between concussions during a sports career and long-term neurodegenerative conditions, such as dementia.^[6]^ Also, scientific evidence shows that retired professional rugby players are associated with small to moderate impairments in cognitive function (e.g. attention, memory, and concentration) when compared with age-matched controls.^[7]^ This is even more so among retired professional rugby players with a significant history of career-related concussions.

The health challenges likely to occur after a career in professional rugby are related to diverse health domains and thus warrant a holistic approach for the support of retired players. These health challenges also recognise that professional rugby stakeholders have a duty to care for retired players.

## Duty of care in professional rugby

As stated by the World Health Organization (WHO) and the International Labour Organization (ILO), ‘protection, promotion, surveillance and maintenance of the highest degree of physical, mental and social well-being of workers in all occupations long after they enter their retirement years’ is a fundamental human right that should be facilitated by social partners and stakeholders. Logically, professional rugby stakeholders have a duty to care, protect and promote the long-term health of retired players. In professional football, the concept of ‘exit health examination’ has been advanced, while the After Career Consultation was recently developed in order to empower the sustainable physical, mental and social health, and the quality of life of retired professional footballers.^[8,9]^ During qualitative research conducted using questionnaires and semi-structured interviews among eight Dutch retired professional footballers (mean age: 34 years; mean career duration: 13 years), the after career consultation revealed a number of medical conditions (e.g. heart rhythm disturbances, knee complaints with limited range of motion and signs of arthrosis) that triggered specific referral and/or guidance from medical practitioners.^[9]^ Even more so, the relevancy, suitability and added value of this support were positively evaluated by the retired professional footballers.^[9]^ Therefore, the after career consultation is globally implemented in the professional football industry by the Fédération Internationale de Football Association (FIFA: governing body) and Football Players Worldwide (FIFPRO: international players union). Analogously to football, and because of the unequivocal duty of care stakeholders have towards retired players, support measures should also be developed and implemented in professional rugby.

## After Rugby Career Consultation (ARCC)

Based on scientific evidence and according to the needs of active and retired players, the ARCC was developed to empower the sustainable health and quality of life of retired professional rugby players. The ARCC relies on information from the following three sources: (1) educational material, (2) medical examination, and (3) guidance, referral or monitoring (if needed).

The educational material consists of semi-medical information related to eight health domains that are relevant for players (e.g. detraining, injuries, OA, nutrition, mental health symptoms) and interviews with elite athletes who share their tips and tricks towards various challenges (including lifestyle). The educational material also includes information on how to prevent the occurrence or worsening of several health conditions in players. The medical examination is based on self-report measures (e.g. validated questionnaires for mental health symptoms), a thorough medical history (e.g. medication use, concussion history), a physical examination (e.g. blood pressure, clinical OA) and several assessments (e.g. standard resting 12-lead electrocardiogram, cognitive functions). The medical examination is based on a standardised protocol (Box 1), ideally by a sports medicine physician with extended experience in professional rugby. Based on all the information gathered, potential health challenges are then discussed with the players. Depending on the findings from the medical examination, specific advice and/or guidance is provided. If needed, the retired professional rugby player might be referred for additional assessment(s) to a medical specialist (e.g. cardiologist, psychiatrist). In the two years following the medical examination, the health of the retired professional rugby player is monitored while potential additional needs are explored.[Table t1-2078-516x-33-v33i1a10651]

Box 1 **After Rugby Career Consultation: overview of the medical examination**

**Table t1-2078-516x-33-v33i1a10651:** 

**Characteristics** Age; Marital situation; Level of education; Current study; Number of studying hours; Current employment; Number of working hours; Smoking status; Lifestyle (e.g., level of physical activity, nutrition); Health-related quality of life
**Rugby history** Playing position; Duration of rugby career; Number of matches played; Level of play; Duration of retirement
**Medical history** Hospitalisation; Diseases; Family history; Medication use; Musculoskeletal severe injuries; Surgeries; Concussions
**Mental health symptoms** Anxiety; Depression; Sleep disturbance; Alcohol misuse; Drug misuse
**Physical examination** Height; Weight; Body fat percentage; Resting heart rate; Blood pressure; Pulmonary function; Musculoskeletal status (e.g. range of motion of joints, signs of overuse tendon insertions, active and passive stability); Clinical osteoarthritis
**Additional assessments** Standard resting 12-lead electrocardiogram; Cognitive functions (e.g. memory, attention)

There is no fixed post-career window to provide retired professional rugby players with the services of ARCC Some players might need support in the first months after they retire from professional rugby, while some others might be facing challenges later in their transitioning years. However, it is important to note that the ARCC also acts preventatively in that it provides retired professional rugby players with educational material and advice on how to remain healthy, promoting a healthy lifestyle and the prevention of the occurrence or worsening of health conditions. Therefore, players who recently retired from professional rugby might be the primary target group rather than older players who might have already digested their transition out of the sport. Rugby is a team sport and analogously, the professional rugby stakeholders within a given country should work together as a team to facilitate the ARCC.

## Piloting the ARCC in South Africa

As with other high-speed collision sports, professional rugby has been increasingly under scrutiny when it comes to the long-term health of players. In order to contribute to the further development of their approach to this, the South African Rugby Union (SARU) and the South African Rugby Legends Association (SARLA) have allied to pilot the ARCC within the context of South African rugby. This will provide an insight into its feasibility and whether retired professional rugby players are satisfied with such a unique support measure. The hope is that such insight will guide future decisions in South Africa as well as the possibility of implementing the ARCC globally. There is no time to waste: a step towards necessary health support for retired professional rugby players is needed now.
